# Genetics of Hemostasis: Differential Effects of Heritability and Household Components Influencing Lipid Concentrations and Clotting Factor Levels in 282 Pediatric Stroke Families

**DOI:** 10.1289/ehp.10754

**Published:** 2008-02-21

**Authors:** Ulrike Nowak-Göttl, Claus Langer, Sandra Bergs, Sabine Thedieck, Ronald Sträter, Monika Stoll

**Affiliations:** 1 Department of Pediatric Hematology/Oncology; 2 Institute of Clinical Chemistry/Laboratory Medicine, and; 3 Leibniz Institute for Arteriosclerosis Research, University of Münster, Münster, Germany

**Keywords:** heritability, household, lifestyle, pediatric stroke, smoking

## Abstract

**Background:**

The identification of heritable and environmental factors possibly influencing a condition at risk should be a prerequisite for the search for the proportion of variance attributable for shared environmental effects (c^2^) modulating the risk of disease. Such epidemiologic approaches in families with a first acute ischemic stroke during early childhood are lacking.

**Objectives:**

Our goal was to estimate the phenotypic variation within lipid concentrations and coagulation factor levels and to estimate the proportions attributable to heritability (h^2^r) and c^2^ in pediatric stroke families.

**Methods:**

Blood samples were collected from 1,002 individuals from 282 white stroke pedigrees. We estimated h^2^r and c^2^ for lipoprotein (a) [Lp(a)], cholesterol, high-density lipoprotein, low-density lipoprotein (LDL), fibrinogen, factor (F) II, FV, FVIIIC, von Willebrand factor (vWF), antithrombin, protein C, protein S, plasminogen, protein Z, total tissue factor pathway inhibitor (TFPI), prothrombin fragment F1.2, and D-dimer, using the variance component method in sequential oligogenetic linkage analysis routines.

**Results:**

When incorporating h^2^r and c^2^ in one model adjusted for age, blood group, sex, smoking, and hormonal contraceptives, significant h^2^r estimates were found for Lp(a), LDL, fibrinogen, protein C, and protein Z. In addition to the significant h^2^r estimates, c^2^ showed a significant effect on phenotypic variation for fibrinogen, protein C, and protein Z. A significant c^2^ effect was found for cholesterol, and plasma levels of FII, FV, vWF, antithrombin, protein S, plasminogen, and TFPI, ranging from 9.3% to 33.2%.

**Conclusions:**

Our research stresses the importance of research on the genetic variability and lifestyle modifications of risk factors associated with pediatric stroke.

Numerous clinical and environmental conditions result in elevated thrombin generation with subsequent thrombus formation not only in adults but also in children (Andrew et al. 1994; Schmidt and Andrew 1995). Both genetic and environmental factors have been established as causes of cardiovascular disease (CVD)—for example, coronary heart disease, stroke, and deep venous thrombosis (DVT) (Edwards et al. 1999; Stephens and Humphries 2003). Stroke in children is a rare disease with an estimated incidence of 2.6 per 100,000 per year (Schoenberg et al. 1978), with half of the events reported presenting as acute ischemic strokes (AISs). Risk factors of AIS in children include congenital heart malformations, vascular abnormalities, endothe-lial damage, infectious diseases, and collagen tissue diseases, as well as some rare inborn metabolic disorders (Kirkham et al. 2000; Nicolaides and Appelton 1996). In addition, it has recently been demonstrated that hyper-coagulable states associated with *a*) the presence of the factor (F) V G1691A mutation, the FII G20210A variant, *b*) increased concentrations of lipoprotein(a) [Lp(a)], and *c*) deficiency states of antithrombin, protein C, protein S, and tissue factor pathway inhibitor (TFPI) represent risk factors for AIS in childhood (Düring et al. 2004; Haywood et al. 2005; Israels and Seshia 1987; Nowak-Göttl et al. 1999a, 1999b; Sträter et al. 2002). In addition, measurable risk factors for CVD in adults further include traits such as obesity, high blood pressure, elevated serum cholesterol, and low levels of high-density lipoprotein (HDL), with an aggregation within families (Gardner et al. 1996; Lamarche et al. 1998; Stampfer et al. 1996). These studies suggest that genetic factors are important in determining CVD. In adult cohorts, however, there is increasing evidence that in addition to genetic risk factors influ-encing lipid and coagulation factor levels, modifiable environmental factors such as smoking, alcohol consumption, diet, or exercise are likely to contribute to the pathogenesis of CVD (Czerwinski et al. 2004; Middelberg et al. 2002; Mosher et al. 2005; Perusse et al. 1997). Developing statistical methodology allows investigation of traits whose susceptibility to familial influences impinges on the risk of diseases at interest (Almasy and Blangero 1998). The identifica-tion of heritable and environmental factors possibly influencing a condition at risk should be a prerequisite for the search for *a*) quantitative trait loci affecting such traits and *b*) household effects modulating the risk of disease. Thus far, such epidemiologic approaches in various population cohorts of different size—for example, healthy twins (Ariens et al. 2002; de Lange et al. 2001; Heller et al. 1993; Middelberg et al. 2006; Snieder et al. 1997), Spanish idiopathic thrombosis families (Souto et al. 2000), relatives of protein C–deficient pedigrees (Vossen et al. 2004), or parent–offspring pairs from national health surveys (Freeman et al. 2002; Saunders and Gulliford 2006)—have been performed mainly in adult cohorts. Although these studies included pediatric offspring, they did not focus primarily on pediatric disease. To date, such studies in families with first onset of stroke during early childhood are lacking. Because estimates for heritability may provide insights into the relative importance of genetic and environmental variables associated with AIS in children, the present study was conducted.

## Methods

### Ethics

The present study was performed in accordance with the ethical standards laid down in the updated version of the 1964 Declaration of Helsinki (World Medical Association 2002) and was approved by the medical ethics committee of the University of Münster, Germany. With written parental consent, term neonates and children with confirmed diagnosis of AIS who were ≤ 18 years of age at onset, biological brothers and sisters, and available parents (nuclear family) were enrolled.

### Study population and study design

From July 1996 to August 2006, 1,002 household members of 282 white pediatric index patients enrolled in the Münster stroke database were analyzed (Düring et al. 2004; Nowak-Göttl et al. 1999b; Sträter et al. 2002). AIS was con-firmed by standard imaging methods—duplex sonography, magnetic resonance imaging, and computerized tomography, and/or magnetic resonance angiography (Nowak-Göttl et al. 1999b; Sträter et al. 2002). All family members enrolled were personally interviewed regarding their medical history, surgery, trauma, immobilization, infections, pregnancies, and use of any medication such as oral contraceptives, hormone replacement therapy, antibiotics, lipid-lowering therapy, and antihypertensive or antidiabetic drugs. In addition, data on objectively confirmed thromboembolic events including age at onset, triggering factors, imaging methods performed, and use of antithrombotic/antiplatelet therapy and its duration were documented. Preterm AIS infants and patients > 18 years of age at first AIS onset were not included in the present study. In addition, patients with suspected AIS without the diagnosis being confirmed by an independent experienced pediatric neuroradiologist were excluded from the present survey. Seven adult patients using vitamin K antagonists were excluded from the analysis for vitamin K–dependent coagulation proteins, D-dimer, and prothrombin F1.2. In addition, we excluded 18 parents from the analysis for lipid measurements who were taking lipid-lowering medication.

### Blood sample collection

We collected blood samples from patients and relatives in the morning after a 12-hr fasting period (for infants, 4–6 hr); samples were drawn by peripheral venipuncture into plastic tubes containing 1/10 by volume of 3.8% trisodium citrate (Sarstedt, Nümbrecht, Germany) and were immediately placed on melting ice. Serum plastic tubes purchased from Sarstedt were used to collect samples to measure lipid components. The blood samples from patients were collected 6–12 months after the acute thrombotic event and at least 6 weeks apart from anticoagulation. Platelet-poor plasma or serum was prepared by centrifugation at 3,000 × *g* and 4°C for 2 × 20 min, aliquoted in polystyrene tubes, stored at –70°C, and thawed immediately before assay. DNA extraction was performed by a spin column procedure (QIAGEN GmbH, Hilden, Germany) as previously described (Junker et al. 1999). For the present study, blood samples were drawn from otherwise healthy patients, relatives, and controls with normal hemograms and no evidence of further diseases.

### Laboratory analyses

With written parental consent, laboratory analyses including Lp(a), kringle 4 repeats, total cholesterol, low-density lipoprotein (LDL), HDL, the FV G1691A mutation, the FII G20210A variant, fibrinogen, FV, chromogenic FII, FVIIIC, von Willebrand factor (vWF) antigen, antithrombin, protein C, free protein S, protein Z, plasminogen, total TFPI, D-dimer, prothrombin fragment f1.2 (f1.2), and antiphospholipid antibodies/lupus anticoagulants were performed as previously described (Heller et al. 2003; Junker et al. 1999; Kreuz et al. 2006; Nowak-Göttl et al. 1999a, 1999b; Ravi et al. 1998). Based on the availability of appropriately stored serum and plasma samples, the number of individuals per assay varied from 582 to 1,002.

### Statistics

Statistical analyses were performed using the StatView 5 (SAS Institute Inc., Cary, NC, USA) and SOLAR software packages (Almasy and Blangero 1998). Because nonparametric tests are less dependent on the shape of the underlying distributions, continuous data are presented as medians (minimum–maximum) and are evaluated statistically by using the Wilcoxon–Mann–Whitney *U* test or Kruskal–Wallis test, corrected for multiple testing according to Bonferroni. On the assumption that multiple genes with small effects influence pediatric AIS, as well as the premise of having normally distributed traits, we estimated heritability (h^2^r) for lipid concentrations and coagulation factor levels using the variance component method implemented in SOLAR. This method allows the total phenotypic variance to be partitioned into a proportion caused by polygenic effects and a proportion caused by random environmental effects, including the proportion of variance in a trait that can be attributed to common environmental factors/ household effects (c^2^). The calculated h^2^r thus estimates the total variance of a trait explained by additive genetic effects. Before variance component analysis, we tested the phenotypic distribution using the Kolmogorov–Smirnov test to assure normal distribution of the trait within the population, as required for parametric analytic procedures. Traits failing the requirements of normality in the Kolmogorov–Smirnov test were transformed through a logarithmic transformation and retested for normalcy. All traits were normally distributed except for Lp(a), which was subsequently logarithmically transformed and then passed the Kolmogorov–Smirnov test for normalcy. To adjust for covariates possibly explaining the phenotypic h^2^r or c^2^ variation in this pediatric AIS family study, we entered the FV G1691A mutation, the prothrombin G20210A variant, age at blood sample collection, blood group, sex, smoking, and the use of oral contraceptives, into the final model of the variance component analysis. Both variance components and covariates were estimated simultaneously by maximum likelihood techniques as implemented in SOLAR. We tested the null hypothesis of no genetic effect (h^2^r = 0) on phenotype with a likelihood ratio test by comparing the likelihood of a restricted model in which parameter h^2^r was constrained to a value of 0 with that for a general model in which the same parameter was estimated. Standard errors were calculated as part of the iterations preceding the variance component analysis. Because our samples were ascertained via index patients, we conditioned the likelihood of a family on a phenotype of the initial proband to account in part for the nonrandom sampling (Comuzzie and Williams 1999) as implemented in SOLAR. We calculated prevalence rates of prothrombotic risk factors in patients and family members by descriptive statistics and compared them by chi-square analysis or by Fisher’s exact test, if appropriate. The criterion for statistical significance was set at α = 0.05. *p*-Values were based on two-sided test.

## Results

### Study population

One thousand two subjects were recruited and examined from 282 pedigrees and households including 498 children; the structure of pedigrees varied from 3 to 10 individuals, with a median of three family members sharing one household. The main characteristics of the 1,002 family members are shown in Table 1. The median age of the index patients was 4 years, and the whole population, of which 50.6% members were male, had a median age of 22 years. Whereas sex, body mass index (BMI), and the proportion of prothrombotic risk factors did not differ significantly between the offspring groups, age at blood sample collection and the rate of smoking was significantly increased in parents. Table 2 describes median (minimum–maximum) values of the lipid concentrations and coagulation factors investigated. Again, there was no statistical difference between the pediatric groups tested. In contrast, as expected, median (minimum–maximum) values of cholesterol, LDL, FII, protein C, protein Z, and plasminogen were significantly higher in parents compared with their offspring.

### Heritability analysis

When we measured h^2^r without accounting for possible environmental effects, h^2^r estimates were highly significant for lipid concentrations and coagulation factor levels investigated except for F1.2. The significant proportion of phenotypic variance explained by h^2^r ranged from 11.8% for cholesterol to 78.8% for Lp(a) kringle 4 repeats [Supplemental Material, Table 1 (online at http://www.ehponline.org/members/2008/10754/suppl.pdf)].

### Heritability analysis including c^2^ estimate

When incorporating h^2^r and c^2^ in one model adjusted for modifiers, such as the FV G1691A mutation, the FII G20210A variant, age at blood sample collection, blood group, sex, smoking, and the use of oral contraceptives in females, we found high and significant h^2^r estimates for Lp(a) serum levels, Lp(a) kringle 4 repeats, LDL, fibrinogen, protein C, and protein Z, with a corresponding phenotypic variance explained by genes of 84.3, 76.5, 24.3, 22.5, 17.7, and 42.3% respectively. In addition to the significant h^2^r estimates, c^2^ showed significant effects on phenotypic variation for fibrinogen (11.7%), protein C (20.1%), and protein Z (30.2%). A significant c^2^ effect without evidence for heritability was found for cholesterol (16.3%) and plasma levels of FII (20.8%), FV (9.3%), antithrombin (30.0%), protein S (33.2%), plasminogen (20.3%), TFPI (24.8%), and vWF (15.8%). Of note, the proportion of phenotypic variance explained by smoking alone was 4% for cholesterol, LDL, and protein S. For most of the traits under study, the model with the best fit was the household polygenic model, except for Lp(a) (polygenic model), antithrombin, D-dimer, FII, and FV (household model). The sporadic model was the best fit model to analyze prothrombin fragment 1.2. In Table 3, we summarize h^2^r, c^2^ estimates, and the role of modifiers if applicable for all lipid concentrations and coagulation factors measured.

## Discussion

The present study was performed to identify heritability and environmental factors possibly influencing AIS in white children and their families. This is the first large-scale family study of the genetics of quantitative variation in putative risk factors associated with AIS in a cohort of children and their young parents. The highest estimates were found for measures of Lp(a) and protein Z, and lower but still significant heritabilities were seen for LDL, fibrinogen, and protein C. Our findings are in accordance with previous data obtained predominantly in adults investigated for h^2^r and c^2^ incorporated in one model (Middelberg et al. 2002; Snieder et al. 1997; Vossen et al. 2004). In addition to the h^2^r estimates mentioned previously, in the present survey a significant concomitant c^2^ effect was found for protein Z, protein C, and fibrinogen. We confirmed a shared c^2^ effect for levels of vWF, which has previously been shown by Vossen et al. (2004) in a large pedigree of a protein C–deficient family.

Elevated Lp(a) plays a role for first AIS in white children and also represents a risk factor for early stroke recurrence in this cohort (Nowak-Göttl et al. 1999b; Sträter et al. 2002). Based on the literature, heritability estimates of Lp(a) are age independent (Middelberg et al. 2006; Snieder et al. 1997). Our finding is in line with the observation that Lp(a) in Europeans is determined in most cases by a single gene located on chromosome 6q26-27 (Scholz et al. 1999), identified as the major quantitative trait locus, and that at least a second locus, recently reported (Broeckel et al. 2002), might be involved. Vossen et al. (2004) reported on high heritability estimates for protein Z (66.7%), with an environmental effect of 6.6%. In our study, however, based mainly on the different study design, h^2^r for protein Z was lower—for example, 42.3%, with a shared c^2^ estimate of 30.2%. Although the information on protein Z heritability estimates was congruent, contradictory data have been reported for protein Z plasma levels associated with stroke in adults. On one hand, AIS was associated with low protein Z concentrations (Vasse et al. 2001); on the other hand, elevated protein Z concentrations were associated with AIS in adults (Kobelt et al. 2001; Lichy et al. 2004; Staton et al. 2005). Because the protein Z phenotypes are influenced not only by genes but also by acute-phase reactions (McQuillan et al. 2003), the identifica-tion of additive genetic effects influencing the variability of this phenotype will contribute to the future understanding of the role of protein Z in adult or pediatric AIS.

The main difference of our study from previous reports is based on the additional estimation of c^2^ effects: Here we present an important influence of the shared c^2^ estimates on phenotypic variation explained by genes adjusted for FV G1691A, FII G20210A, age at blood sample collection, blood groups, sex, smoking, use of hormonal contraceptives for cholesterol, and plasma levels of FII, FV, antithrombin, protein S, plasminogen, and TFPI, with significant estimates ranging from 9.3% for FV to 33.2% for protein S. One reason for the contradictory results relating to plasma levels of FII and FV, respectively, might be the fact that in our analysis FV G1691A and FII G20210A variants were introduced as covariates in the analysis.

In our family study we included 498 children ≤ 18 years of age with a median age of 5 years, which means that the duration of the parent–offspring pair relationships was at least 5 years. Because our population differs in age and the source of CVD—pediatric AIS—from the studies previously published (Ariens et al. 2002; Beekman et al. 2002; de Lange et al. 2001; Heller et al. 1993; Middelberg et al. 2002, 2006; Perusse et al. 1997; Saunders and Gulliford 2006; Snieder et al. 1997; Souto et al. 2000; Vossen et al. 2004), our data strengthen the hypothesis that except for Lp(a) concentrations, age at investigation as well as different underlying CVDs play a role with respect to the proportion of phenotypic variance explained by additive genetic effects (Heller et al. 1993; Snieder et al. 1997). The young AIS cohort presented here and collected in the Münster pediatric stroke database, including index children, brothers, and sisters as well as parents (nuclear families), offers the unique opportunity for longitudinal subject follow-up, scheduled at a 5-year interval to obtain additional information on genetic and environmental variations in this AIS cohort.

In addition to the attributed magnitude of the c^2^ effect and the age at recruitment, the study design chosen also influences results obtained from family-based surveys. The study populations reported so far differed not only by geographic enrollment but also by sample size and subject selection. Twin studies were performed for lipid measurements by Heller et al. (1993), Snieder et al. (1997), and Middelberg et al. (2002, 2006), and heritability estimates for coagulation factors in healthy twins were measured by de Lange et al. (2001) and Ariens et al. (2002) in the United Kingdom. Souto et al. (2000) estimated heritability in family members of probands with idiopathic thrombophilia in Spain, and Vossen et al. (2004) enrolled relatives from a large protein C–deficient family. Subject recruitment from healthy families in the United Kingdom was performed by Freeman et al. (2002), and a small, two-generation pedigree study from the U.K. national health survey was recently published by Saunders and Gulliford (2006). Table 4 summarizes studies measuring h^2^r as well as c^2^ (Ariens et al. 2002; de Lange et al. 2001; Mitchell et al. 1996; Saunders and Gulliford 2006; Souto et al. 2000; Vossen et al. 2004). Thus, we suggest that the lower heritability reported in the present two-generation pedigrees of young stroke children, their parents, and siblings, with a median of three nuclear subjects per household compared with other studies, might be explained mainly by age and AIS as CVD origin.

A limitation of such a study is that a measurement error can be associated with decreased heritability estimates, as discussed by Souto et al. (2000). The lower heritability estimates presented here, however, are unlikely to be caused by measurement error, as the intraassay coefficients of variation (ICV) and run-to-run coefficients of variation (RCV) reported for the methodologies used were small, for example, ranging from 1.3% (vWF) to 6.2% (F1.2) for ICV and 3.3% (FVIIIC) to 9.5% (F1.2) for RCV, respectively (Kreuz et al. 2006). In addition, data reported here are limited to the cohort investigated—that is, white German stroke children. As outlined, our family-based study sample consists of nuclear families with one or more first-degree siblings (no half-siblings) and their parents who were ascertained via index patients. To partly accommodate the nonrandom sampling, we conditioned our analyses on the phenotype of the initial proband (Comuzzie and Williams 1999). However, we cannot rule out that the reported estimates for h^2^r and c^2^ may be overfit and are not representative of the general population.

In conclusion, in addition to the strong heritability estimates found for Lp(a) kringle 4 repeats and Lp(a) concentrations and, to a lesser extent, for protein Z, LDL, fibrinogen, and protein C, our findings strengthen the importance of shared environmental influences of lipids and levels of coagulation factors during childhood and early adulthood, hereby pointing to the potential for family-based lifestyle interventions. Along with the diagnostic workup of known prothrombotic polymorphisms associated with stroke onset in white German children (Düring et al. 2004; Nowak-Göttl et al. 1999a, 1999b; Sträter et al. 2002), the main focus on AIS studies in this cohort will include future research to identify novel genes involved in the control of quantitative trait loci, as well as environmental risk factors with a strong c^2^ effect (such as smoking) as the main factors essential in prevention of AIS in children. Families with affected subjects are advised to change their lifestyle to reduce their CVD risk for future offspring.

## Figures and Tables

**Table 1:** Pedigree characteristics (index children, biological siblings, and parents).

Characteristic	AIS children (*n* = 282)	Siblings (*n* = 216)	Parents (*n* = 504)
Disease/health status			
AIS/DVT/MI (no.)	282/—/—	3/—/—	1/8/3
Age (years) at blood collection [median (min–max)]	4 (0.1–18)	6 (0.1–18)	35 (17–65)*
Male sex [no. (%)]	152 (54)	115 (53)	241 (47.9)
BMI (kg/m^2^) [median (min–max)]	16.0 (7.9–30.9)	17.4 (10.8 –29.3)	24.6 (17.7–46.9)*
Risk factors			
FVG1691A [no. (%)]	41 (14.5)	23 (10.6)	59 (11.7)
Prothrombin G20210A [no. (%)]	19 (6.7)	6 (2.8)	18 (3.6)
Antithrombin-/protein C–/protein S–deficiency/APS (no.)	0/4/0/4	0/0/0/0	0/4/0/1
Lp(a) > 30 mg/dL [no. (%)]	72 (25.5)	53 (24.4)	131 (26.0)
Smoking > 12 years of age [no. (%)]	3 (1.1)	10 (4.6)	77 (15.3)*
Use of oral contraceptives [no. (%)]	—	6 (2.8)	31 (11.8)
Therapy			
Aspirin/vitamin K antagonists (no.)	25/—	—/—	3/7
Antihypertensive/antidiabetic/lipid-lowering therapy (no.)	4/0/0	0/0/0	75/5/18

Abbreviations: —, no event; max, maximum; MI, myocardial infarction; min, minimum.

* Compared with children, *p* < 0.05.

**Table 2:** Median (min–max) values of lipid and coagulation factor levels in index patients and relatives.

Characteristic	Individuals tested (no.)	AIS children *(n* = 282)	Siblings (*n* = 216)	Parents (*n* = 504)
Lipid components [median (min–max)]				
Lp(a) (mg/dL)	1,002	14 (0–168)	16 (0.6–126)	18 (1–201)
Lp(a) kringle 4 repeats	971	27 (12–37)	25 (13–37)	27 (8–37)
Total cholesterol (mg/dL)	870	161 (91–249)	160 (96–222)	192 (93–323)*
LDL (mg/dL)	868	85 (24–177)	87 (38–171)	111 (22–236)*
HDL (mg/dL)	870	55 (25–104)	58 (29–96)	59 (27–111)
Coagulation factors [median (min–max)]				
Fibrinogen (mg/dL)	1,002	247 (128–572)	248 (36–528)	260 (136–572)
FII (%)	1,002	98 (24 –172)	98 (18–164)	107 (62–201)*
FV (%)	1,002	100 (25–195)	105 (13–1074)	111 (50–182)
FVIIIC (%)	934	102 (39–192)*	105 (59–184)	118 (34–236)
vWF (% )	870	103 (19–279)	102 (25–220)	109 (38–281)
Antithrombin (%)	820	103 (71–131)	107 (66–136)	103 (74–150)
Protein C (%)	960	89 (23–196)	91 (18–174)	109 (39–200)*
Protein S (%)	960	94 (42–162)	90 (38–156)	94 (35–188)
Protein Z (μg/mL)	582	1.3 (0.21–8)	1.4 (0.44–3.2)	1.6 (0.33–4.9)*
Plasminogen (%)	934	94 (44–135)	96 (49–151)	105 (62–207)*
TFPI (ng/mL)	865	57 (21–116)	40 (28–113)	47 (19–116)
D-dimer (mg/L)	752	0.16 (0–0.6)	0.18 (0–2.0)	0.12 (0–1.6)
Prothrombin F1.2 (nmol/L)	826	0.8 (0–6.5)	0.7 (0.1–4.5)	0.8 (0–8.1)

Abbreviations: max, maximum; min, minimum.

* Compared with children, *p* < 0.05.

**Table 3:** Proportion of phenotypic variance explained by covariates (%), h^2^r, and c^2^ (% ± SE).

Factor of interest	Covariates (%)	h^2^r ± SE	*p*-Value	c^2^ ± SE	*p*-Value^a^
Lp(a)	R	84.3 ± 9.5	< 0.0001	0.4 ± 6.5	0.5
Lp(a) kringle 4 repeats	R	76.5 ± 8.5	< 0.0001	8.0 ± 6.7	0.11
Cholesterol (total)	A/S (23.5)	9.3 ± 1.3	0.23	16.3 ± 6.5	0.006
HDL	A/G/S (10.0)	15.7 ± 14.6	0.14	9.4 ± 7.2	0.1
LDL	A/G/S (16.8)	24.3 ± 13.1	0.034	10.2 ± 6.6	0.060
Fibrinogen	A/G (2.5)	22.5 ± 12.7	0.038	11.7 ± 6.4	0.035
FII	A/G/FII (11.8)	0	0.5	20.8 ± 3.5	0.0001
FV	A (0.4)	0	0.5	9.3 ± 2.3	0.002
FVIIIC	A/G (3.5)	5.9 ± 1.8	0.37	12.1 ± 8.3	0.07
vWF	FII (0.001)	23.4 ± 16.1	0.075	15.8 ± 8.1	0.024
Antithrombin	A (2.6.)	0	0.5	30.0 ± 3.8	< 0.0001
Protein C	A (12.2)	17.7 ± 10.7	0.049	20.1 ± 5.6	0.0002
Protein S	A/G/S (15.2)	14.9 ± 11.6	0.099	33.2 ± 6.3	< 0.0001
Protein Z	A/G/H (1.6)	42.3 ± 11.0	0.0001	30.2 ± 8.5	0.0002
Plasminogen	A/G/S/FII (6.6)	18.2 ± 11.9	0.07	20.3 ± 6.4	0.0008
TFPI	A/G	0	0.5	24.8 ± 5.5	0.009
D-dimer	FV/G/S (5.3)	0	0.5	7.5 ± 3.6	0.13
Prothrombin fragment F1.2	R	0	0.5	R	—

R, removed from the model.

a Adjusted for age (A), FII G20210A (FII), FV G1691A (FV), sex (G), use of oral contraceptives (H), and smoking (S).

**Table 4:** Comparison of h^2^r and c^2^ estimates.

Our study	SAFHS (Mitchell et al. 1996)		Gait (Souto et al. 2000)		United Kingdom (Ariens et al. 2002; De Lange et al. 2001)		Vermont (Vossen et al. 2004)		United Kingdom (Saunders and Gullifort 2006)		Münster (Nowak-Göttl et al. 2008)	

Households/pedigrees/ individuals (no.)	655/42/1,236		153/21/397		491 twin pairs		181/1/322		6,183/—/17,690		282/282/1,002	
Age [years (min–max)]	16–94		37.7 (< 1–88)		18–79/21–73		31.3 (1–90)		Adults > 16 years		22 (0.1–65)	
CVD	Healthy community- based cohort		Idiopathic DVT > 45 years		Community-based twins		Protein C–deficient family		Healthy community- based cohort		Pediatric stroke cohort	
Ethnicity	Mexican American		White Spanish		White		White French Canadian		White (1,308 nonwhite)		White German	
Cohort	h^2^r	c^2^	h^2^r	c^2^	h^2^r	c^2^	h^2^r	c^2^	h^2^r	c^2^	h^2^r	c^2^
Lp(a)	69*	5	—	—	—	—	—	—	—	—	84.3*	0.4
Cholesterol	39*	4	—	—	—	—	—	—	40*	8*	9.3	16.3*
HDL	45*	2	—	—	—	—	—	—	—	—	15.7*	9.4
LDL	40*	7	—	—	—	—	—	—	—	—	24.3*	10.2
Fibrinogen	—	—	33.6*	13.7*	44	0	29.7*	0	23	16*	22.5*	11.7*
FII	—	—	49.2*	0	57	0	70*	4.1	—	—	0	20.8*
FV	—	—	44*	13*	—	—	71.4*	2.8	—	—	0	9.3*
FVIIIC	—	—	40*	0	—	—	—	—	—	—	5.9	12.1
vWF	—	—	31.8*	0	75	0	25.3*	30.7*	—	—	23.4	15.8*
Antithrombin	—	—	48.6*	0	—	—	6	33.5*	—	—	0	30.0*
Protein C	—	—	50*	0	—	—	40.6*	4.4	—	—	17.7*	20.1*
Protein S	—	—	22*	21*	—	—	10.5	37*	—	—	14.9	33.2*
Protein Z	—	—	—	—	—	—	67*	6.6	—	—	42.3*	30.2*
Plasminogen	—	—	23.6*	0	—	—	—	—	—	—	18.2	20.3*
TFPI	—	—	51.6*	0	—	—	—	—	—	—	0	24.8*
D-dimer	—	—	10.9	0	65	—	7	5.2	—	—	0	7.5
Prothrombin fragment F1.2	—	—	—	—	45	—	22*	44*	—	—	0	R

Abbreviations: —, no data; max, maximum; min, minimum; R, removed from the model; SAFHS, San Antonio Family Heart Study.

* *p* < 0.05.

## References

[b1-ehp0116-000839] Almasy L, Blangero J (1998). Multipoint quantitative trait linkage analysis in general pedigrees. Am J Hum Genet.

[b2-ehp0116-000839] Andrew M, David M, Adams M, Ali K, Anderson R, Barnard D (1994). Venous thromboembolic complications (VTE) in children: first analyses of the Canadian registry of VTE. Blood.

[b3-ehp0116-000839] Ariens RAS, de Lange M, Snieder H, Boothby M, Spector TD, Grand PJ (2002). Activation markers of coagulation and fib-rinolysis in twins: heritability of the prethrombotic state. Lancet.

[b4-ehp0116-000839] Beekman M, Heijmans BT, Martin NG, Pedersen NL, Whitfield JB, DeFaire U (2002). Heritabilities of apolipoprotein and lipid levels in three countries. Twin Res.

[b5-ehp0116-000839] Broeckel U, Hengstenberg C, Mayer B, Holmer S, Martin LJ, Comuzzie AG (2002). A comprehensive linkage analysis for myocardial infarction and its related risk factors. Nat Genet.

[b6-ehp0116-000839] Comuzzie AG, Williams JT (1999). Correcting for ascertainment bias in the COGA data set. 1999. Genet Epidemiol.

[b7-ehp0116-000839] Czerwinski SA, Mahaney MC, Rainwater DL, Vandeberg JL, MacCluer JW, Stern MP (2004). Gene by smoking interaction: evidence for effects on low-density lipoprotein size and plasma levels of triglyceride and high-density lipoprotein cholesterol. Hum Biol.

[b8-ehp0116-000839] de Lange M, Snieder H, Ariens RAS, Spector TD, Grant PJ (2001). The genetics of haemostasis: a twin study. Lancet.

[b9-ehp0116-000839] Düring C, Kosch A, Langer C, Thedieck S, Nowak-Göttl U (2004). Total tissue factor pathway inhibitor is an independent risk factor for symptomatic paediatric venous thrombosis and stroke. Thromb Haemost.

[b10-ehp0116-000839] Edwards KL, Mahaney MC, Motulsky AG, Austin MA (1999). Pleiotropic genetic effects on LDL size, plasma triglyc-eride, and HDL cholesterol in families. Arterioscler Thromb Vasc Biol.

[b11-ehp0116-000839] Freeman MS, Mansfield MW, Barrett JH, Grant PJ (2002). Genetic contribution to circulating levels of hemostatic factors in healthy families with effects of known genetic polymorphisms on heritability. Arterioscler Thromb Vasc Biol.

[b12-ehp0116-000839] Gardner CD, Fortmann SP, Krauss RM (1996). Association of small low-density lipoprotein particles with the incidence of coronary artery disease in men and women. JAMA.

[b13-ehp0116-000839] Haywood S, Liesner R, Pindora S, Ganesan V (2005). Thrombo-philia and first arterial ischaemic stroke: a systematic review. Arch Dis Child.

[b14-ehp0116-000839] Heller C, Heinecke A, Junker R, Knöfler R, Kosch A, Kurnik K (2003). Cerebral venous thrombosis in children: a multifactorial origin. Circulation.

[b15-ehp0116-000839] Heller DA, de Faire U, Pedersen NL, Dahlen G, McClearn GE (1993). Genetic and environmental influence on serum lipid levels in twins. N Engl J Med.

[b16-ehp0116-000839] Israels SJ, Seshia SS (1987). Childhood stroke associated with protein C or S deficiency. J Pediatr.

[b17-ehp0116-000839] Junker R, Koch HG, Auberger K, Münchow N, Ehrenforth S, Nowak-Göttl U (1999). Prothrombin G20210A gene mutation and further prothrombotic risk factors in childhood thrombophilia. Arterioscler Thromb Vasc Biol.

[b18-ehp0116-000839] Kirkham FJ, Prengler M, Hewes KM, Ganesan V (2000). Risk factors for arterial ischemic stroke in children. J Child Neurol.

[b19-ehp0116-000839] Kobelt K, Biasiutti FD, Mattle HP, Lämmle B, Wuillemin A (2001). Protein Z in ischaemic stroke. Br J Haematol.

[b20-ehp0116-000839] Kreuz W, Stoll M, Junker R, Heinecke A, Heinecke A, Schobess R (2006). Familial elevated factor VIII in children with symptomatic venous thrombosis and postthrombotic syndrome. Results of a multicentre study. Arterioscler Thromb Vasc Biol.

[b21-ehp0116-000839] Lamarche B, Tchernof A, Mauriege P, Cantin B, Dagenais GR, Lupien PJ (1998). Fasting insulin and apolipoprotein B levels and low-density lipoprotein particle size as risk factors for ischemic heart disease. JAMA.

[b22-ehp0116-000839] Lichy C, Kropp S, Dong-Si T, Genius J, Dolan T, Hampe T (2004). A common polymorphism of the protein Z gene is associated with protein Z plasma levels and with risk of cerebral ischemia in the young. Stroke.

[b23-ehp0116-000839] McQuillan AM, Eikelboom JW, Hankey GJ, Baker R, Thom J, Staton J (2003). Protein Z in ischemic stroke and its etiologic subtypes. Stroke.

[b24-ehp0116-000839] Middelberg RP, Martin NG, Whitfield JB (2006). Longitudinal genetic analysis of plasma lipids. Twin Res Hum Genet.

[b25-ehp0116-000839] Middelberg RPS, Spector TD, Swaminathan R, Snieder H (2002). Genetic and environmental influences on lipids, lipopro-teins and apolipoproteins. Effects of menopause. Arterioscler Thromb Vasc Biol.

[b26-ehp0116-000839] Mitchell BD, Kammerer CM, Blangero J, Mahaney MC, Rainwater DL, Dyke B (1996). Genetic and environmental contributions to cardiovascular risk factors in Mexican Americans. The San Antonio Family Heart Study. Circulation.

[b27-ehp0116-000839] Mosher MJ, Martin LJ, Cupples LA, Yang Q, Dyer TD, Williams JT (2005). Genotype-by-sex interaction in the regulation of high-density lipoprotein: the Framingham Heart Study. Hum Biol.

[b28-ehp0116-000839] Nicolaides P, Appelton RE (1996). Stroke in children. Dev Med Clin Neurol.

[b29-ehp0116-000839] Nowak-Göttl U, Junker R, Hartmeier M, Koch HG, Münchow N, Assmann G (1999a). Increased lipoprotein (a) is an important risk factor for venous thrombosis in childhood. Circulation.

[b30-ehp0116-000839] Nowak-Göttl U, Sträter R, Heinecke A, Junker R, Koch HG, Schuierer G (1999b). Lipoprotein (a) and genetic polymorphisms of clotting factor V, prothrombin, and methylenetetrahydrofolate reductase are risk factors of spontaneous ischaemic stroke in childhood. Blood.

[b31-ehp0116-000839] Perusse L, Rice T, Despres JP, Bergeron J, Province MA, Gagnon J (1997). Familial resemblance of plasma lipids, lipoproteins and postheparin lipoprotein and hepatic lipases in the HERITAGE family study. Arterioscler Thromb Vasc Biol.

[b32-ehp0116-000839] Ravi S, Mauron T, Lämmle B, Wuillemin WA (1998). Protein Z in healthy human individuals and in patients with a bleeding tendency. Br J Haematol.

[b33-ehp0116-000839] Saunders CL, Gulliford MC (2006). Heritabilities and shared environmental effects were estimated from household clustering in national health survey data. J Clin Epidemiol.

[b34-ehp0116-000839] Schmidt B, Andrew M (1995). Neonatal thrombosis: report of a prospective Canadian and international registry. Pediatrics.

[b35-ehp0116-000839] Schoenberg B, Mellinger J, Schoenberg D (1978). Cerebro-vascular disease in infants and children: a study of incidence, clinical features, and survival. Neurology.

[b36-ehp0116-000839] Scholz M, Kraft HG, Lingenhel A, Delport R, Delport R, Vorster EH (1999). Genetic control of lipoprotein(a) concentrations is different in Africans and Caucasians. Eur J Hum Genet.

[b37-ehp0116-000839] Snieder H, van Doornen LJP, Boomsma DI (1997). The age dependency of gene expression for plasma lipids, lipopro-teins, and apolipoproteins. Am J Hum Genet.

[b38-ehp0116-000839] Souto JC, Almasy L, Borrell M, Gari M, Martinez E, Mateo J (2000). Genetic determinants of hemostasis phenotypes in Spanish families. Circulation.

[b39-ehp0116-000839] Stampfer MJ, Krauss RM, Ma J, Blanche PJ, Holl LG, Sacks FM (1996). A prospective study of triglyceride level, low-density lipoprotein particle diameter, and risk of myocardial infarction. JAMA.

[b40-ehp0116-000839] Staton J, Sayer M, Hankey GJ, Cole V, Thom J, Eikelboom JW (2005). Protein Z gene polymorphisms, protein Z concentrations, and ischaemic stroke. Stroke.

[b41-ehp0116-000839] Stephens JW, Humphries SE (2003). The molecular genetics of cardiovascular disease: clinical implications. J Intern Med.

[b42-ehp0116-000839] Sträter R, Becker S, von Eckardstein A, Heinecke A, Gutsche S, Junker R (2002). Prospective assessment of risk factors for recurrent stroke during childhood—a 5-year follow-up study. Lancet.

[b43-ehp0116-000839] Vasse M, Guegan-Massardier E, Borg JY, Woimant F, Soria C (2001). Frequency of protein Z deficiency in patients with ischaemic stroke. Lancet.

[b44-ehp0116-000839] Vossen CY, Hasstedt SJ, Rosendaal FR, Callas PW, Bauer KA, Broze GJ (2004). Heritability of plasma concentrations of clotting factors and measurements of a prethrombotic state in a protein C-deficient family. J Thromb Haemost.

[b45-ehp0116-000839] World Medical Association (2002). Declaration of Helsinki. http://www.wma.net/e/policy/b3.htm.

